# Astaxanthin Supplemented with High-Intensity Functional Training Decreases Adipokines Levels and Cardiovascular Risk Factors in Men with Obesity

**DOI:** 10.3390/nu15020286

**Published:** 2023-01-06

**Authors:** Ayoub Saeidi, Akbar Nouri-Habashi, Omid Razi, Ali Ataeinosrat, Hiwa Rahmani, Shirin Shirzad Mollabashi, Behnam Bagherzadeh-Rahmani, Shahin Mahmoudi Aghdam, Leila Khalajzadeh, Maisa Hamed Al Kiyumi, Anthony C. Hackney, Ismail Laher, Katie M. Heinrich, Hassane Zouhal

**Affiliations:** 1 Department of Physical Education and Sport Sciences, Faculty of Humanities and Social Sciences, University of Kurdistan, Sanandaj 66177-15175, Iran; 2 Department of Exercise Physiology and Corrective Movements, Faculty of Sport Sciences, Urmia University, Urmia 57561-51818, Iran; 3 Department of Exercise Physiology, Faculty of Physical Education and Sports Science, Razi University, Kermanshah 94Q5+6G3, Iran; 4 Department of Physical Education and Sport Science, Science and Research Branch, Islamic Azad University, Tehran 14778-93855, Iran; 5 Faculty of Physical Education and Sports Science, Alzahra University, Tehran 19938 93973, Iran; 6 Faculty of Sport Sciences, Atatürk University, Erzurum 25030, Turkey; 7 Department of Exercise Physiology, Faculty of Sport Sciences, Hakim Sabzevari University, Sabzevar M3J+373, Iran; 8 Department of Exercise Physiology, Central Tehran Branch, Islamic Azad University, Tehran 14778-93855, Iran; 9 Department of Family Medicine and Public Health, Sultan Qaboos University Hospital, Muscat H5QC+36M, Oman; 10 Department of Exercise & Sport Science, University of North Carolina, Chapel Hill, NC 27599, USA; 11 Department of Nutrition, University of North Carolina, Chapel Hill, NC 27599, USA; 12 Department of Anesthesiology, Pharmacology and Therapeutics, The University of British Columbia, Vancouver, BC V6T 1Z4, Canada; 13 Department of Kinesiology, College of Health and Human Sciences, Kansas State University, Manhattan, KS 66506, USA; 14 Laboratoire Mouvement, Sport, Santé, University of Rennes, M2S—EA 1274, 35000 Rennes, France; 15 Institut International des Sciences du Sport (2I2S), 35850 Irodouer, France

**Keywords:** high-intensity training, astaxanthin, adipokines, cardiovascular risk factors, obesity

## Abstract

The aim of this study was to investigate the effects of 12 weeks of high-intensity training with astaxanthin supplementation on adipokine levels, insulin resistance and lipid profiles in males with obesity. Sixty-eight males with obesity were randomly stratified into four groups of seventeen subjects each: control group (CG), supplement group (SG), training group (TG), and training plus supplement group (TSG). Participants underwent 12 weeks of treatment with astaxanthin or placebo (20 mg/d capsule daily). The training protocol consisted of 36 sessions of high-intensity functional training (HIFT), 60 min/sessions, and three sessions/week. Metabolic profiles, body composition, anthropometrical measurements, cardio-respiratory indices and adipokine [Cq1/TNF-related protein 9 and 2 (CTRP9 and CTRP2) levels, and growth differentiation factors 8 and 15 (GDF8 and GDF15)] were measured. There were significant differences for all indicators between the groups (*p* < 0.05). Post-hoc analysis indicated that the levels of CTRP9, CTRP2, and GDF8 were different from CG (*p* < 0.05), although levels of GDF15 were similar to CG (*p* > 0.05). Levels of GDF8 were similar in the SG and TG groups (*p* > 0.05), with reductions of GDF15 levels in both training groups (*p* < 0.05). A total of 12 weeks of astaxanthin supplementation and exercise training decreased adipokines levels, body composition (weight, %fat), anthropometrical factors (BMI), and improved lipid and metabolic profiles. These benefits were greater for men with obesity in the TSG group.

## 1. Introduction

Obesity promotes co-morbid diseases such as cardiovascular diseases, type 2 diabetes, and metabolic syndrome [1,2], and is associated with increased adipocyte size, increased production of reactive oxygen species (ROS), secretion of pro-inflammatory cytokines, and lipid deposition to promote insulin resistance in peripheral tissues [3,4,5,6]. Adipocytes release proteins such as adiponectin, leptin, resistin, and visfatin into the bloodstream [1], in addition to others such as Cq1/TNF-related proteins (CTRPs) and growth differentiation factors (GDFs) [7,8]. CTRPs released by adipose tissue regulate lipid and glucose metabolism [9]. The CTRP adipokine family consists of 15 members (CTRP-1 to CTRP-15) with localized tissue expression, where CTRP-2 and CTRP-9 are highly expressed in adipocytes. The secretion of CTRP-2 and CTRP-9 is increased during obesity in humans and in animal models [10,11], making CTRP-2 and CTRP-9 targets for the medical management of obesity [7,12]. GDFs, including GDF-8 and GDF-15, are secretory proteins of the transforming growth factor (TGF)-β family [13]. GDF-8 or myostatin plays a critical role in skeletal muscle homeostasis and reduced expression of GDF-8 is associated with fat loss, increased insulin sensitivity, and increased glucose uptake [14]. Levels of GDF-15, also known as macrophage inhibitory cytokine (MIC-1) released by adipocytes, positively correlate with obesity [15], with a suggested role in lipid metabolism [16]. Treatments to reduce obesity and its related health problems remain elusive. Pharmacological and surgical approaches in obese patients are associated with side effects and have variable effectiveness [17,18]. Reducing energy consumption, increasing energy expenditure, and increasing muscle mass hold promise in the management of obesity and associated complications [19,20]. However, the effects of regular exercise training to manage obesity and related diseases are related to the modes of exercise [21]. For example, CrossFit training (a high-intensity mixed exercise model of concurrent strength and endurance performance) reduces lipid oxidation in obese individuals [22,23]. This high-intensity functional training (HIFT) exercise modality consists of exercise sets with or without rest times between sets [24] and has increased IL-6 and IL-10 activity [25], elevated aerobic capacity, improved muscular endurance, increased lean body mass, and reduced body fat [26]. Another way to reduce or prevent obesity is to use natural antioxidant supplements. Astaxanthin (3, 3′-dihydroxy-β, β-carotene-4, 4′-dione) is derived from *Haematoccus pluvialis* algae and has health benefits in treating some cancers, chronic inflammatory disease, diabetes, obesity, cardiovascular diseases, and neurodegenerative disorders [27,28]. Astaxanthin reduces the effects of oxidative stress on lipid metabolism [29]. Using astaxanthin as a dietary supplementation can expedite lipid metabolism in muscles during exercise [29]. Despite the well-accepted benefits of exercise training on lipid oxidation and metabolic disorders in obese patients, astaxanthin, as a supplement rich in antioxidants, can also improve metabolism and reduce the inflammation caused by obesity. There is limited information about the benefits of supplementing HIFT with astaxanthin on cardiovascular risk factors and adipokine levels for individuals with obesity. We hypothesized that supplementing astaxanthin with HIFT increases weight loss and attenuates the effects of CTRPs and GDFs and fat loss in individuals with obesity. To this end, we examined the effects of 12 weeks of HIFT supplemented with astaxanthin on body composition, cardio-respiratory fitness, adipokine levels, insulin resistance and lipid profiles in males with obesity.

## 2. Materials and Methods

After calling in public places such as gyms, medical clinics, hospitals and social networks, there were 101 participants who initially volunteered for the study, of whom 33 were ineligible, leaving 68 participants in the study (mean age: 27.6 ± 8.4 yrs; mean height: 167.8 ± 3.1 cm; mean weight: 94.7± 2.0 kg; mean BMI: 33.6 ± 1.4 kg/m^2^), who were divided into 4 groups of 17 per group. The inclusion criteria for the study were: BMI > 30 kg/m^2^, not involved in regular physical activity during the last six months, without endocrine, metabolic and cardiovascular diseases, and without alcohol consumption. Participants taking supplements and medications that may have an effect on adipose and muscle tissue or presenting joint disorders or physical disabilities were also excluded from the study. During the first visit, all participants undertook a physical examination performed by a physician and a clinical exercise physiologist. All the participants provided written informed consent forms and completed a Physical Activity Readiness Questionnaire (PAR-Q) [30]. Study procedures were explained during the first visit and the Research and Ethics Committee of the Islamic Azad University approved all procedures of this study (Ethics code: IR-IAU1400-47). All procedures were performed according to the latest revision of the Declaration of Helsinki [31].

### 2.1. Experimental Design

One week prior to the start of the training programs, the study procedures were explained and a familiarization session was completed by all participants. Height, weight, and body composition were assessed for all the participants, who were then randomly assigned into one of four equal groups (*n* = 17 per group): Control group (CG), Supplement group (SG), Training group (TG), and Training + supplement group (TSG). During the experiment period, 8 participants from different groups withdrew from the study due to medical reasons, work-related difficulties and no interest to continue the research, leaving 15 participants in each group. During the third session, body composition and VO_2peak_ were determined and instructions on how to perform the training programs were given to each group. The two training groups (TG and TSG) performed the exercise training program for 12 weeks, 3 sessions/week. The CG was instructed to not change their current lifestyles during the experiment. All the measurements were performed at the same time of day (within ~1 h) and under the same environmental conditions (~20 °C and ~55% humidity). Baseline assessments were obtained 48 h before the start of the training protocols and post-test measurements were performed 48 h after the last session in all groups.

### 2.2. Body Composition and Cardio-Respiratory Fitness Assessments

Body weights and heights were measured using a calibrated scale (Seca GmbH & Co., Hamburg, Germany) and stadiometer (Seca GmbH & Co., Hamburg, Germany), respectively. Then, the body mass index (BMI, kg/m^2^) was calculated. A bio-impedance analyzer (Medigate Company Inc., Gyeonggi-do, Korea) was used to determine fat-free mass (FFM) and fat mass (FM). In a temperature-controlled room (21–23 °C), a modified Bruce protocol [32,33] using an electronically motorized treadmill (H/P/Cosmos, Pulsar med 3p- Sports and Medical, Nussdorf-Traunstein Germany) allowed for measuring VO_2peak_. The criteria used to determine VO_2peak_ were based on the American College of Sports Medicine (ACSM) guidelines [34]. During this test, blood pressure was measured using an electronic sphygmomanometer (Kenz BPM AM 300P CE, Suzuken Company, Nagoya, Japan), and heart rate was monitored with a Polar V800 heart monitor (Kempele, Finland). Gas analysis was performed using a gas analyzer system (Metalyzer 3B analyzer, Cortex: biophysik, GMbH, Leipzig, Germany) which was calibrated before each test.

### 2.3. Training Protocols

In the current study, a total of 36 sessions lasting up to 60 min each were included in the HIFT program using CrossFit. Crossfit training is stratified as high-intensity functional training with a mixed model of concurrent strength and endurance performance often containing various fitness components [26]. This modality of training consists of some workout sets that may or may not include rest between sets [25]. This type of training is associated with some physiological effects, such as increased blood lactate [35,36], elevated testosterone levels, increased cortisol [37], and increased IL-6 and IL-10 activity [25], as well as some adaptations in elevating aerobic capacity, muscular endurance, lean body mass, utilization of body fat or reducing body fat [26]. All HIFT sessions were led by a trainer with a Level 1 CrossFit certificate according to the methods described earlier [38,39]. Average times for each workout of the day (WOD) and total average WOD time per week were calculated for the HIFT group as a whole (i.e., participants in the TG and TSG).

### 2.4. Supplementation of Astaxanthin and Placebo

Eligible participants in the SG and TSG received 20 mg/day of astaxanthin capsule (Marine Product Tech. Inc., Seongnam, Republic of Korea) or matching placebo capsule (20 mg/day of raw corn starch) once daily with breakfast for 12 weeks. Participants were considered adherent when ≥80% of their prescribed supplements were consumed.

### 2.5. Nutrient Intake and Dietary Analysis

Before and after the experiment changes in habitual dietary intake over time were determined using three-day food records (two weekdays and one weekend day) [40]. Nutritional intakes were analyzed using Diet Analysis Plus version 10 (Cengage, Boston, MA, USA) (Table 1).

### 2.6. Assessment of Blood Markers

Fasting blood samples were taken under standard conditions between 8 and 10 am from the right arm 12 h before the first exercise session and 72 h after the last session. Blood samples were transferred to EDTA-containing tubes, centrifuged for 10 min at 3000 rpm, and stored at −70 °C for later use for the following measurements:

Plasma CTRP-9 was measured with an ELISA kit (Aviscera Bioscience, Santa Clara, CA, USA). Catalogue No: SK00081-02. Sensitivity: 1 ng/mL. Intra-coefficients of variation (CV) = 4%, inter-CV = 8%.

CTRP-2 was measured using ELISA kits obtained from MyBioSource (San Diego, CA, USA) with a minimum detectable dose (MDD) of 0.039 ng/mL and detection range of 0.156–10 ng/mL (intra-assay CV: <8% and inter-assay < 10%).

GDF-15 was measured using ELISA kits obtained from Thermo Scientific (Frederick, MD, USA) with a sensitivity of 2 pg/mL and detection range of 1.10–800 pg/mL (intra-assay CV < 10% and inter-assay CV < 12%).

Plasma GDF8 was measured with an ELISA kit (R&D Systems, Minneapolis, MN, USA) Catalogue No: DGDF80. The sensitivity was 5.32 pg/mL. Intra-CV = 5.4%, inter-CV = 6%.

Plasma total cholesterol (TC) and triglyceride (TG) were measured by enzymatic methods (CHOD-PAP). High-density cholesterol (HDL-C) and low-density cholesterol (LDL-C) were determined using a photometric method (Pars Testee’s Quantitative Detection kit, Tehran, Iran) with a coefficient and sensitivity of 1.8% and 1 mg/dL and 1.2% and 1 mg/dL, respectively.

Insulin levels were measured with an ELISA kit (Demeditec, Kiel, Germany) with a sensitivity of 1 ng/mL and between and within CV of 5.1% and 8.4%, respectively. 

Glucose levels were measured with a colorimetric enzymatic kit (Paracetamol Glucose, Colorimetric Enzymatic kit, Parsazmun, Tehran, Iran) with a sensitivity of 5 mmol/L. 

HOMA-insulin resistance (HOMA-IR) was calculated from the ratio of insulin to glucose and the HOMA-IR index [HOMA-IR = fasting insulin (mU/L) × glucose (mmol/L)/22.5] [41].

### 2.7. Statistical Analysis

Descriptive statistics (means ± standard deviation) were used to summarize all data. The normality of the data was assessed by the Shapiro–Wilk test. A two-way ANOVA repeated measures test was used to determine group X time interactions. One-way ANOVA and Fisher LSD post-hoc tests were used for evaluation of baseline data for the four groups. When a significant difference was detected by ANOVA, mean differences were determined by pairwise comparisons. The sample size needed to detect a statistical difference between study variables with a 95% confidence interval (CI) and equal to or greater than 80% of the power value was calculated. Effect sizes (ES) were reported as partial eta-squared, where ES were considered trivial (<0.2), small (0.2–0.6), moderate (0.6–1.2), large (1.2–2.0) and very large (2.0–4.0). A *p*-value of <0.05 was used to indicate statistical significance. All data were analyzed using SPSS software (version 24; Armonk, NY, USA).

## 3. Results

### 3.1. Anthropometric Characteristics and Cardio-Respiratory Parameters

There were no between-group differences in baseline values for weight (*p* = 0.46), BMI (*p* = 0.57), body fat (*p* = 0.33), FFM (*p* = 0.59), or VO_2_peak (*p* = 0.98). There were significant group X time interactions for weight (*p* = 0.0001, η^2^ = 0.46), BMI (*p* = 0.002, η^2^ = 0.30), fat percent (*p* = 0.0001, η^2^ = 0.51), FFM (*p* = 0.0001, η^2^ = 0.43), and VO_2_peak (*p* = 0.0001, η^2^ = 0.61) (Table 2). Body weight reductions after 12 weeks were significant in the SG (*p* = 0.008), TG (*p* = 0.0001), and TSG (*p* = 0.0001) but not in the CG (*p* = 0.32). Furthermore, the post-hoc test for body weight shows that after 12 weeks there were significant changes in the CG compared to the TG (*p* = 0.004) and TSG (*p* = 0.0001), and in the TSG compared to the TG (*p* = 0.01) and SG (*p* = 0.0001), while other changes were not significant (*p* > 0.05) (Table 2).

Changes in BMI after 12 weeks were significantly decreased in the SG (*p* = 0.019), TG (*p* = 0.0001) and TSG (*p* = 0.0001) but not in the CG (*p* = 0.37). BMI changes after 12 weeks were significantly decreased in the TG (*p* = 0.016) and the TSG (*p* = 0.0001) compared to the CG. The differences induced by training were significant between the TG and TSG (*p* = 0.007) and between the SG and TSG (*p* = 0.007), while all other differences in BMI between the groups were not significant (*p* > 0.05) (Table 2). Increases in post-test values of FFM were significant in comparison to pre-test values in the SG (*p* = 0.0001), TG (*p* = 0.0001) and TSG (*p* = 0.0001) but not for the CG (*p* = 0.08). Between-group differences in FFM were significant in the CG compared to the SG (*p* = 0.0001), TG (*p* = 0.0001) and TSG (*p* = 0.0001) but all other between-group differences were not significant (*p* > 0.05) (Table 2). The decrease in body fat percent after 12 weeks was significant in the SG (*p* = 0.004), TG (*p* = 0.0001) and TSG (*p* = 0.0001) but not in the CG (*p* = 0.28). Between-group differences in body fat percent were significant in the CG compared to the SG (*p* = 0.006), TG (*p* = 0.0001) and TSG (*p* = 0.0001), and also between the SG and TSG (*p* = 0.013), while all other differences were not significant (*p* > 0.05) (Table 2). Increases in the VO_2_peak after 12 weeks of exercise were significant in the TG (*p* = 0.0001) and TSG (*p* = 0.0001) but not in the CG (*p* = 0.32) and SG (*p* = 0.21). Between-group differences in VO_2_peak were significant in the CG compared to the TG (*p* = 0.0001) and TSG (*p* = 0.0001), and also in the SG compared to the TG (*p* = 0.0001) and TSG (*p* = 0.0001), while all other differences were not significant (*p* > 0.05) (Table 2).

### 3.2. Lipid Profiles

There were no significant differences in the baseline levels of HDL (*p* = 0.57), LDL (*p* = 0.71), TC (*p* = 0.17), and TGs (*p* = 0.47) in the four groups. There were significant group X time interactions for HDL (*p* = 0.0001, η^2^ = 0.89), LDL (*p* = 0.0001, η^2^ = 0.86), TC (*p* = 0.0001, η^2^ = 0.95), and TGs (*p* = 0.0001, η^2^ = 0.95) (Table 2). Post-test HDL levels increased in the SG (*p* = 0.0001), TG (*p* = 0.0001) and TSG (*p* = 0.0001) groups compared to pre-test values but were unchanged in the CG (*p* = 0.88). Bonferroni’s post-hoc test showed that increases in HDL after 12 weeks of training were significant in all groups (*p* = 0.0001) except for differences between the TG and TSG (*p* = 0.06) (Table 2). Post-test levels of LDL decreased in the SG (*p* = 0.0001), TG (*p* = 0.0001) and TSG (*p* = 0.0001) but not in the CG (*p* = 0.82). Differences in LDL after 12 weeks of training were significant among all groups (*p* = 0.0001) except for differences between the TG and TSG (*p* = 0.39) (Table 2). Post-test levels of TC decreased in the SG (*p* = 0.0001), TG (*p* = 0.0001) and TSG (*p* = 0.0001) but not in the CG (*p* = 0.88). Differences in TC after 12 weeks of training were significant in all groups (*p* = 0.0001) (Table 2). Post-test levels of TGs were reduced in the SG (*p* = 0.0001), TG (*p* = 0.0001) and TSG (*p* = 0.0001) but not in the CG (*p* = 0.47). Differences in TGs after 12 weeks of training were significant in all groups (*p* = 0.0001) except for differences between the TG and TSG (*p* = 0.64) (Table 2).

### 3.3. Metabolic Factors

There were no significant differences in baseline levels of glucose (*p* = 0.66), insulin (*p* = 0.53), and HOMA (*p* = 0.49) in the four groups. There were significant group X time interactions for glucose (*p* = 0.0001, η^2^ = 0.49), insulin (*p* = 0.0001, η^2^ = 0.78) and HOMA (*p* = 0.0001, η^2^ = 0.69) (Table 2). Glucose levels decreased significantly in the SG (*p* = 0.0001), TG (*p* = 0.0001), and TSG (*p* = 0.0001), yet did not significantly change in the CG (*p* = 0.06). Post-hoc tests revealed significant between-group differences (*p* = 0.0001) except for the CG and SG (*p* = 0.056) and the TSG and TG (*p* = 0.26) (Table 2). Insulin levels significantly decreased in the SG (*p* = 0.0001), TG (*p* = 0.0001), and TSG (*p* = 0.0001), with no significant changes in the CG (*p* = 0.21). Post-hoc between-group differences were significant for all groups (*p* = 0.0001) (Table 2). Levels of HOMA-IR decreased following 12 weeks of training in the SG (*p* = 0.0001), TG (*p* = 0.0001), and TSG (*p* = 0.0001), while the difference in HOMA-IR in the CG was not significant (*p* = 0.17). Post-hoc between-group differences were significant for all groups (*p* = 0.0001) except for the TG and TSG (*p* = 0.065) (Table 2).

### 3.4. Adipokines and Growth Differentiation Factors

There were no significant differences in baseline levels of CTRP9 (*p* = 0.22), CTRP2 (*p* = 0.18), GDF8 (*p* = 0.75) and GDF15 (*p* = 0.59) between the four study groups. There were statistically significant group X time interactions for CTRP9 (*p* = 0.001, η^2^ = 0.62), CTRP2 (*p* = 0.0001, η^2^ = 0.71), GDF8 (*p* = 0.0001, η^2^ = 0.38) and GDF15 (*p* = 0.0001, η^2^ = 0.75).

A comparison of pre-test and post-test values indicated no differences in CTRP9 (*p* = 0.30), CTRP2 (*p* = 0.30), GDF8 (*p* = 0.30) and GDF15 (*p* = 0.30) for CG, the result was also not significant in the SG for GDF15 (*p* = 0.07). Differences from the pre-test to post-test were that GDF15 was significantly lower in the TG (*p* = 0.0001) and TSG (*p* = 0.0001), SG (*p* = 0.0001), and CTRP9, GDF8 and GDF15 were significantly lower in the TG (*p* = 0.0001) and TSG (*p* = 0. 0.0001).

**Figure 1:**
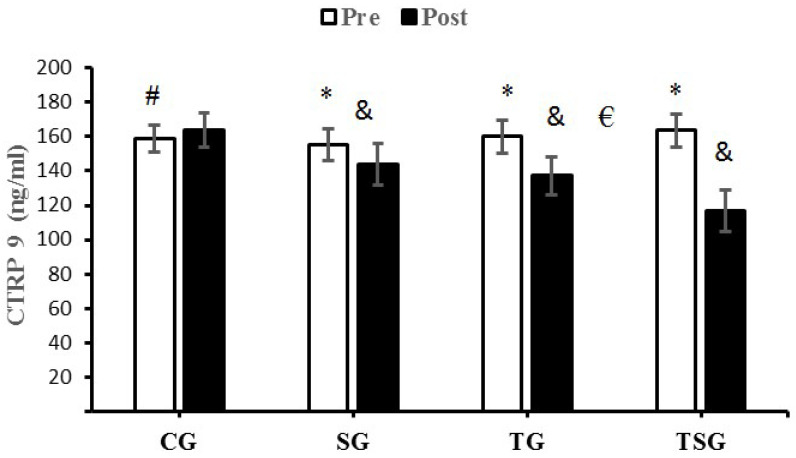
Pre- and post-training values (mean ± SD) for CTRP9 in the control (CG), supplement (SG), training (TG), training + supplement (TSG) groups. ^&^ Significant differences with pretest values (*p* < 0.05). * Significant differences with the control group (*p* < 0.05). ^#^ Significant interaction between time and group (*p* < 0.05). ^€^ Significant time X group interaction (*p* < 0.05).

The analysis of between-group differences for CTRP9 indicated a significance in TG (*p* = 0.0001) and TSG (*p* = 0.0001) compared to the CG. The decrease in CTRP9 in the TSG following 12 weeks of training was significantly greater than the decreases in the SG (*p* = 0.0001) and TG (*p* = 0.001) (Figure 1).

**Figure 2:**
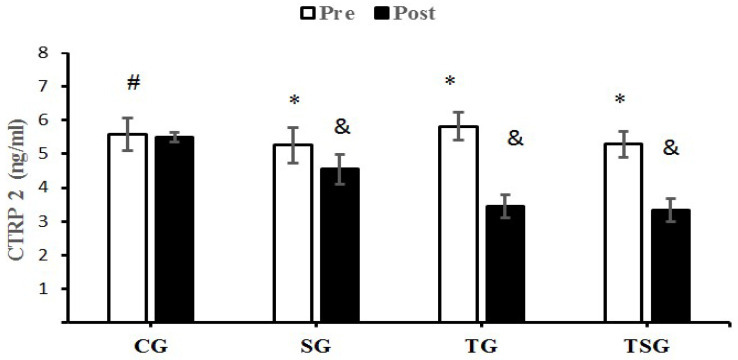
Pre- and post-training values (mean ± SD) for CTRP2 in the control (CG), supplement (SG), training (TG), training + supplement (TSG) groups. ^&^ Significantly different from pretest values (*p* < 0.05). * Significantly different from control group (*p* < 0.05). ^#^ Significant time X group interaction (*p* < 0.05).

The decreases of CTRP2 in the SG (*p* = 0.024), TG (*p* = 0.0001) and TSG (*p* = 0.0001) were significantly greater than the changes in the CG but the differences between the TG and TSG were not significant (*p* = 0.11). The decrease in CTRP2 in TG (*p* = 0.0001) and TSG (*p* = 0.001) were significantly different from the changes in the SG following 12 weeks of training (Figure 2). 

**Figure 3:**
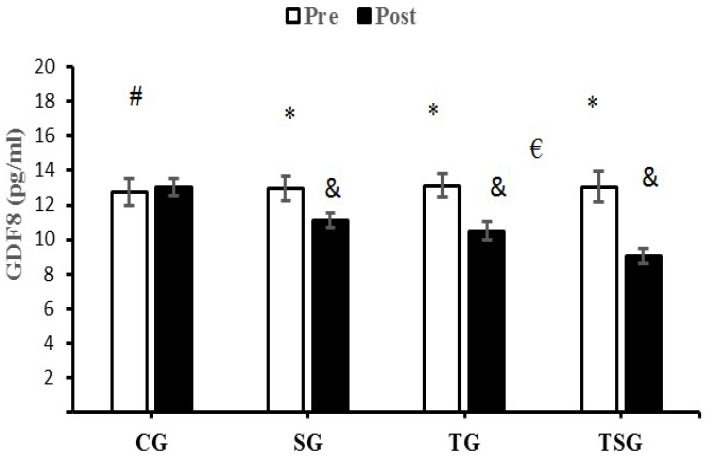
Pre- and post-training values (mean ± SD) for GDF8 in the control (CG), supplement (SG), training (TG), training + supplement (TSG) groups. ^&^ Significantly different from pretest values (*p* < 0.05). * Significantly different from CG (*p* < 0.05). ^#^ Significant interaction between time and group (*p* < 0.05). ^€^ Significant time X group interaction (*p* < 0.05).

GDF8 decreased significantly in the SG (*p* = 0.0001), TG (*p* = 0.0001) and TSG (*p* = 0.0001) compared to the CG. Additionally, Bonferroni’s post-hoc test showed that the decrease in GDF8 in TSG following 12 weeks of training was different from the changes in TG (*p* = 0.001) and SG (*p* = 0.0001) (Figure 3).

**Figure 4:**
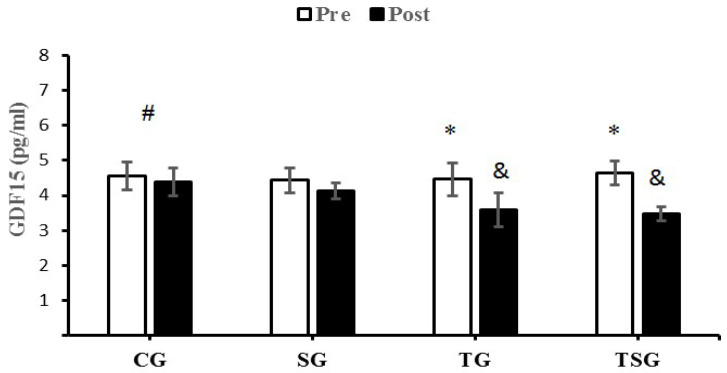
Pre- and post-training values (mean ± SD) for GDF15 in the control (CG), supplement (SG), training (TG), training + supplement (TSG) groups. ^&^ Significantly different from pretest values (*p* < 0.05). * Significantly different from CG (*p* < 0.05). ^#^ Significant time X group interaction (*p* < 0.05).

GDF15 significantly decreased in the TG (*p* = 0.004) and TSG (*p* = 0.0001) compared to the CG. Additionally, Bonferroni’s post-hoc test showed that the decrease in GDF15 in TG (*p* = 0.02) and TSG (*p* = 0.001) following 12 weeks of training were significantly different from the SG (Figure 4).

## 4. Discussion

We examined the effects of 12 weeks of HIFT that was supplemented with astaxanthin on adipokine levels, insulin resistance and lipid profiles in males with obesity. Our findings indicate that 12 weeks of CrossFit exercise training supplemented with astaxanthin: (A) reduced anthropometric indices (body weight, BMI, FFM, body fat percent), (B) improved cardio-respiratory fitness (measured by VO_2_peak), (C) improved lipid profiles (HDL-C LDL-C, TC, and TGs), (D) improved post-test metabolic markers (glucose, insulin, and HOMA-IR, and (E) reduced adipokine (CTRP2, CTRP9) and growth differentiation factor (GDF8, and GDF15) levels. Obesity is associated with increased adipocyte hypertrophy and hyperplasia. Increased fat accumulation by adipocytes is accompanied by the inhibition of lipolysis and promotion of lipogenesis by inhibiting mitochondrial β-oxidation [42], AMPK activity, and increasing the activity of enzymes involved in lipogenesis [43,44]. An accumulation of fat in the liver, skeletal muscle, and adipocytes leads to insulin resistance (IR) by activating protein kinase C θ (PKC θ) [45]; IR increases fatty acid mobilization from adipose tissue to circulation [46] and triggers oxidative-stress mediated inflammation [47]. Adipocyte hypertrophy increases macrophage accumulation and the production of proinflammatory M1 phenotypes that damages pancreatic β-cells [48,49]. The combined effects of these changes increase body weight, FFM, BMI, and exacerbate insulin resistance and related metabolic disorders. Antioxidants such as astaxanthin can improve dyslipidemia and reduce metabolic disorders [50,51]. Our study indicates that HIFT, supplemented astaxanthin and exercise supplemented with astaxanthin reduced circulatory lipid levels and markers of metabolic disorders, and improved body composition and HDL-C levels. This is supported by other findings that astaxanthin prevents body weight gain and improves lipid profiles and fatty acid utilization related to its anti-oxidative and anti-inflammatory properties [52,53]. Astaxanthin increases plasma total antioxidant capacity (TAC) and superoxide dismutase (SOD) levels [54]. Changes in inflammatory conditions may be another pathway through which astaxanthin can improve metabolic changes induced by obesity, as astaxanthin inhibits the activation of the transcriptional factor of NF-κB to reduce the actions of pro-inflammatory cytokines [55]. Additionally, astaxanthin reduces the infiltration of inflammatory M1 macrophages into hypertrophied adipocytes, which mitigates the release of pro-inflammatory cytokines by macrophages and reduces the release of free fatty acids into circulation and improves insulin sensitivity [56,57]. Astaxanthin accelerates fatty acid oxidation during physical exercise [29,58]. Our study showed that the percentage of body fat in the SG and TG decreased, and this improvement was greater in the TSG. Notably, the significant differences between TG and TSG may be due to the intensity of exercise. Our finding of increased fat utilization is consistent with previous reports indicating that HIFT reduced body fat levels in active healthy individuals [59,60]. Increased fat reduction induced by this type of training reflects changes in aerobic capacity, as measured by the VO_2_peak in our study, and supported by other studies that CrossFit exercise increases muscle mass and improves insulin sensitivity. Our findings are supported by other reports that HIIT improves insulin sensitivity [61,62]. Adipokine levels correlate positively with adipose tissue levels in obese animal models and humans [11]. The changes in both CTRPs in our study may be related to changes in body weight and lipid profiles [8,11]. Our findings indicate that HIFT and astaxanthin reduced body weight and lipid profiles, while increasing HDL-C levels; these changes were greater when exercise was supplemented with astaxanthin (Figure 1 and Figure 2). In an investigation that used apoE-knocked out mice fed with a high-fat diet, astaxanthin increased the hepatic levels of LDL receptors, reduced HMG-CoA reductase, and increased Sterol regulatory element-binding protein 2 (SREBP-2), which were associated with hypocholesterolemia effects [63]. Another study that utilized two doses of astaxanthin indicated that the level of TG reduced, while HDL-C increased [64]. Our results are supported by other 12-week studies of combined aerobic and resistance training reduced body weight and CTRP5 and CTRP3 levels in obese women [65].

The release of GDF8 is increased in overweight and obese individuals, suggesting a role for GDF8 in the regulation of body fat and total energy metabolism [19]. GDF8 is a potentially negative regulator of skeletal muscle mass [66,67]. Our study found lower circulatory levels of GDF8 to a greater extent in the group receiving both astaxanthin and HIFT training than when either intervention was studied in isolation. Circulating levels of GDF15 are increased with obesity, chronic inflammation [68,69,70,71], and reduced exercise training [72,73]. Contrary to our findings, the majority of other studies report increases in GDF15 levels following exercise in healthy and obese individuals [73,74,75,76], likely due to acute episodes of metabolic and inflammatory stress [77,78]. The conflicting results between our findings (of reduced GDF15 levels following HIFT training) and those of previous studies (of increases in GDF15 levels with exercise) may be related to differences in training modalities [79].

## 5. Conclusions

Our findings indicate that 12 weeks of astaxanthin supplementation and exercise training decreased adipokines levels, body composition (weight, %fat), anthropometrical factors (BMI), and improved lipid and metabolic profiles. These benefits were greater in men with obesity who exercised and used astaxanthin supplementation. 

## Figures and Tables

**Table 1:** Nutritional intakes in the four study groups.

	CG			SG		TG	TSG	
	Pre	Post	Pre	Post	Pre	Post	Pre	Post
Energy (kcal/day)	2260 ± 47	2269 ± 56	2278 ± 101	2149 ± 100	2269 ± 117	2141 ± 117	2273 ± 157	2129 ± 126
CHO (g/day)	281 ± 31.4	283 ± 33.3	279.4 ± 27.1	261 ± 27.5	289 ± 48.6	261 ± 39.2	288 ± 38.6	259 ± 29.1
Fat (g/day)	82.2 ± 11.0	81 ± 9.8	86.5 ± 10.7	75 ± 11.2	83.4 ± 12.4	73.1 ± 11.2	80.8 ± 13.87	70.2 ± 11.3
Protein (g/day)	104 ± 12.0	106 ± 11.3	101 ± 13.5	93 ± 12.6	103 ± 14.8	94 ± 11.7	102 ± 14.5	90 ± 13.5

Data are mean (±SD) values of CG: Control group; SG: Supplement group; TG: Training group; TSG: Training supplement group; CHO: Carbohydrates.

**Table 2:** Mean (±SD) values of glucose, insulin, lipid profile, body composition, and VO_2peak_.

	CG		SG		TG		TSG	
	Pre	Post	Pre	Post	Pre	Post	Pre	Post
Height (cm)	167.5 ± 2.7	-	168.2 ± 3.5	-	167.5 ± 4.1	-	167.7 ± 3.1	-
Weight (kg)	95.3 ± 1.8	92.1 ± 2.1	94.2 ± 2.6	90.1 ± 2.3 ^a^	94.3 ± 0.9	90.1 ± 2.3 ^a,b^	95.1 ± 1.9	88.2 ± 2.3 ^a,b,ab^
Fat percentage (%)	31.1 ± 1.5	31.8 ± 2.1 ^a^	31.1 ± 1.5	29.0 ± 0.7 ^a,b^	31.3 ± 1.4	27.8 ± 0.9 ^a,b^	32.1 ± 1.3	27.6 ± 1.2 ^a,b,ab^
BMI (kg/m^2^)	34.1 ± 2.5	33.7 ± 1.4	33.2 ± 1.4	32.4 ± 1.6 ^a,b^	33.5 ± 1.7	32.1 ± 1.5 ^a,b^	33.8 ± 1.2	31.8 ± 0.6 ^a,b,ab^
FFM (kg)	26.6 ± 1.2	25.5 ± 2.2	26.1 ± 1.8	28.5 ± 0.8 ^a,b^	25.7 ± 1.2	28.4 ± 1.6 ^a,b^	26.1 ± 1.7	29.3 ± 1.2 ^a,b,ab^
VO_2peak_ (mL·kg^−1^·min^−1^)	26.2 ± 2.4	25.9 ± 2.2	26.1 ± 2.8	26.5 ± 2.9 ^a^	26.3 ± 2.3	28.9 ± 1.9 ^a,b^	26.0 ± 2.1	29.1 ± 2.2 ^a,b,ab^
HDL (mg·dL^−1^)	38.3 ± 1.2	37.4 ± 1.30	37.8 ± 1.23	39.8 ± 1.2 ^a^	37.6 ± 1.6	43.5 ± 1.3 ^a,b^	37.5 ± 1.4	44.2 ± 1.1 ^a,b,ab^
LDL (mg·dL^−1^)	127.2 ± 4.4	127.0 ± 4.7	127.5 ± 5.4	123.4 ± 5.2 ^a,b^	128.7 ± 4.3	113.2 ± 2.9 ^a,b^	129.1 ± 3.6	112.5 ± 2.5 ^a,b,ab^
TC (mg·dL^−1^)	228.7 ± 5.2	228.8 ± 5.2	229.4 ± 5.4	224.0 ± 5.1 ^a,b^	232.1 ± 1.6	211.4 ± 2.6 ^a,b^	231.5 ± 5.1	207.2 ± 2.1 ^a,b^.^ab^
TG (mg·dL^−1^)	244.1 ± 4.3	244.8 ± 3.8	247.8 ± 5.9	244.1 ± 5.4 ^a^	246.5 ± 7.4	217.0 ± 5.4 ^a,b^	244.9 ± 5.9	214.8 ± 4.6 ^a,b,ab^
Insulin (ng·mL)^−1^	19.1 ± 0.6	19.4 ± 0.5	19.1 ± 0.7	17.9 ± 0.5 ^a,b^	19.1 ± 0.4	16.4 ± 0.4	19.4 ± 0.4	15.8 ± 0.5 ^a,b,ab^
Glucose (mg·dL^−1^)	100.4 ± 13.1	94.7 ± 6.4	102.9 ± 10.7	88.7 ± 4.5 ^a,b^	103.2 ± 5.7	78.0 ± 5.4	105.6 ± 7.1	75.5 ± 7.7 ^a,b,ab^
HOMA-IR	4.7 ± 0.7	4.5 ± 0.3	4.8 ± 0.4	3.9 ± 0.2 ^a^	4.8 ± 0.2	3.1 ± 0.2	5.0 ± 0.3	2.9 ± 0.3 ^a,b,ab^

CG: Control group; SG: Supplement group; TG: training group; TSG: training+ Supplement group BMI: Body Mass Index; FFM: Fat-Free Mass; HDL: High-density lipoprotein; LDL: Low-density lipoprotein; TC: Total cholesterol; TG: Triglyceride; HOMA-IR: Homeostatic Model Assessment of Insulin Resistance. ^a^ Indicates significant differences compared to the Pre-values (*p* < 0.05). ^b^ Significant differences compared to the control group (*p* < 0.05). ^ab^ Significant interaction between time and groups (*p* < 0.05).

## Data Availability

The data presented in this study are available within the manuscript.
